# Dynamic Japanese Sign Language Recognition Throw Hand Pose Estimation Using Effective Feature Extraction and Classification Approach

**DOI:** 10.3390/s24030826

**Published:** 2024-01-26

**Authors:** Manato Kakizaki, Abu Saleh Musa Miah, Koki Hirooka, Jungpil Shin

**Affiliations:** School of Computer Science and Engineering, The University of Aizu, Aizuwakamatsu 965-8580, Japan; m5271012@u-aizu.ac.jp (M.K.); d8231105@u-aizu.ac.jp (A.S.M.M.); m5261114@u-aizu.ac.jp (K.H.)

**Keywords:** dynamic hand gesture recognition, japanese sign language (JSL), effective feature selection, machine learning, hand skeleton points

## Abstract

Japanese Sign Language (JSL) is vital for communication in Japan’s deaf and hard-of-hearing community. But probably because of the large number of patterns, 46 types, there is a mixture of static and dynamic, and the dynamic ones have been excluded in most studies. Few researchers have been working to develop a dynamic JSL alphabet, and their performance accuracy is unsatisfactory. We proposed a dynamic JSL recognition system using effective feature extraction and feature selection approaches to overcome the challenges. In the procedure, we follow the hand pose estimation, effective feature extraction, and machine learning techniques. We collected a video dataset capturing JSL gestures through standard RGB cameras and employed MediaPipe for hand pose estimation. Four types of features were proposed. The significance of these features is that the same feature generation method can be used regardless of the number of frames or whether the features are dynamic or static. We employed a Random forest (RF) based feature selection approach to select the potential feature. Finally, we fed the reduced features into the kernels-based Support Vector Machine (SVM) algorithm classification. Evaluations conducted on our proprietary newly created dynamic Japanese sign language alphabet dataset and LSA64 dynamic dataset yielded recognition accuracies of 97.20% and 98.40%, respectively. This innovative approach not only addresses the complexities of JSL but also holds the potential to bridge communication gaps, offering effective communication for the deaf and hard-of-hearing, and has broader implications for sign language recognition systems globally.

## 1. Introduction

**Figure 1:**
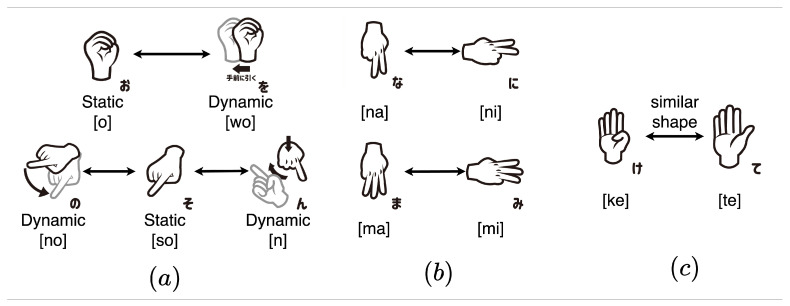
Example of (**a**) The same hand shape, but different meanings depending on the movement (**b**) Same hand shape, but different meanings depending on the angle (**c**) Example of similar shape て (te) and け (ke).

Sign language is an important human communication tool that can be extended to enhance human–computer interaction. It has also contributed to communication at video meetings in recent years between impaired and non-impaired people. Sign language varies from country to country and culture to culture, like other languages. There are many kinds of sign languages in the world. Deaf people use it to establish their communication [1,2,3,4,5,6,7,8]. Since the letters and the way of expression vary from country to country, there are many different sign language patterns. For example, American sign language can express 26 alphabets (A–Z) and ten numbers (1–10). Japanese sign language (JSL) is used in Japan and can express 46 Japanese characters called “Hiragana”. Some sign languages include dynamic finger spelling, and American Sign Language includes two types of J and Z. Because of the many types of letters represented in Japanese Sign Language, five types of finger spelling have movement. There are many deaf and speaking impediment people in Japan [9], and JSL consists of Japanese words that can be constructed either through fingerspelling using hand, finger motion, and hand gestures. In Japanese finger spelling, some static finger spellings are similar in shape to other dynamic finger spellings, making them difficult to classify [1,2,3,4,5]. To express the basic needs of the deaf and speaking impediments, people need to learn JSL, which is not easy for the non-deaf community and needs a human translator, which is costly and not easy. Nowadays, employment attempts for impaired people are promoted in Japan. Three hundred forty thousand people are living in Japan who have speaking or hearing disabilities [9], but many non-disabled people are not able to understand sign language. So, to realize comfortable communication, it is important to recognize sign language by computer. Many researchers have been working to develop sign language recognition using several feature extraction and machine learning-based classification algorithms. To recognize JSL, researchers first extracted various features such mathematical and statistical features such as hand motion, signer motion based on the scale-invariant transforms features, and gradient-based feature extraction [10,11]. They employed principal component analysis (PCA), the Hidden Markov model (HMM), CNNs and DNN algorithm as a classifier [12,13,14,15]. Some other researchers applied support vector machines (SVMs), Artificial neural networks (ANN), Fuzzy classification algorithms (FCA), K-nearest-neighbor (KNN), and AdaBoost algorithm [16,17,18,19]. Ito et al. proposed a CNN to extract features from the JSL dataset, applied SVM for the classification, and achieved 84.20% accuracy [20]. The target is sign words, and as we know, there is a total of 46 JSL sign alphabets. Among them, they are considered static signs and five of them are dynamic or include movement. These five dynamic signs have some moments in which they share the same shape as the example. Figure 1a is an example of dynamic signs in JSL. So, much of Japanese sign language recognition research removes dynamic signs, and their method cannot express these five types of finger spelling that have movement. Most existing systems were developed for static signs by discarding the dynamic alphabet because of the static system. So, much of Japanese sign language recognition research removes dynamic signs, and their method cannot express these five types of finger spelling that have movement. Even if only one character cannot be expressed, many Japanese words become inexpressible and cannot be expressed without using characters represented by dynamic fingers. To overcome the issues, Shiraishi et al. [21] collected the JSL dataset using hand gloves, then applied a machine learning algorithm to classify all 46 Japanese syllabaries, including dynamic signs and obtained 70% accuracy. Also, they included dynamic signs, but their performance is not good, and they are difficult to deploy in real-life applications. Moreover, most of the research work for the JSL recognition domain is related to static JSL recognition, and motion is involved in any existing JSL recognition work. Although a limited number of dynamic JSL recognition work is reported with accuracy lower than 70% [21], as mentioned previously, there are mostly hardware-based and sensor-based approaches, which still have many limitations for deployment in terms of accuracy, portability and cost. Consequently, dynamic JSL recognition is still a challenging problem because of the diversity of the signs from human gestures, arbitrary and multi-views, and dynamic signs from multiple camera viewpoints. It is urgent to solve the problems and increase the performance accuracy of the dynamic JSL recognition task. We proposed a dynamic Japanese sign language recognition with an effective feature and classification approach to solving the existing challenge. In the procedure, we collected the video dataset for JSL and extracted coordinate data with the media pipe. Based on the key points dataset, we proposed four kinds of features that can be derived from that data for recognition and applied classification algorithms. The main contribution of the study is given below:

- **Novelty:** We developed a distinctive JSL video dataset based on dynamic skeletons to fill a crucial void in existing resources. The dataset includes 46 static signs and their dynamic equivalents, gathered from 20 individuals. Through the use of MediaPipe estimation for skeletal data extraction, we effectively tackled challenges such as diverse backgrounds, partial obstruction, computational demands, and varying lighting conditions. This dataset not only addresses a significant research gap but also lays a solid foundation for more comprehensive and inclusive studies in the field of JSL recognition. Although most studies exclude dynamic sign languages, this study includes both dynamic and static sign languages for practical purposes. There are 46 types of signs in Japanese sign language (finger spelling) and five signs include movement. Some signs that have movement share the same shape as other static signs, for example, as in Figure 1.
  There are moments when the five dynamic signs share the same shape. Perhaps due to the difficulty of classification, most Japanese Sign Language recognition research removes dynamic signs, making it impossible to express these five types of dynamic signs. If even one character cannot be expressed, it means that many Japanese words will not be able to be expressed. Therefore, in this research, we target all finger spellings, including both dynamic and static, by adding a feature that captures the changes.
- **Innovative Feature Extraction:** Our feature extraction procedure encompasses distance, angle, variation, finger direction, and motion features, delivering a comprehensive representation of JSL signs. Through an innovative process, a constant number of these features are generated regardless of the number of frames, making classification using machine learning possible. In other words, it is possible to recognize dynamic and static signs in the same process.
- **Optimized Feature Selection and Classification:** Using a feature selection approach based on Random Forest (RF), we identified the most relevant features, thereby optimizing the efficiency and effectiveness of our model. Additionally, we employed a kernels-based Support Vector Machine (SVM) algorithm for classification.
- **Comprehensive Evaluation:** We rigorously assessed the proposed model using our newly generated dynamic JSL dataset and the widely recognized Argentine sign language dataset, LSA64. In both instances, our proposed method demonstrated superior performance compared to existing systems.

The rest of the paper is structured as follows: Related work described in Section 2. Section 3 demonstrates the proposed methodology and explains the dataset, feature values, and classification method. Section 4 describes the results, including the optimal parameter values and a comparison between systems with and without variation features shown Section 5. Finally, Section 6 describes the discussion, and the conclusion section follows this.

## 2. Related Work

Machine learning and deep learning have proved their excellence in various fields, such as EMG, EEG, and MRI data for disease detection, human activity recognition and hand gesture detection [22,23,24]. Many researchers have been working to develop sign language recognition systems using image and Skeleton-based datasets. Researchers used various devices, sensors and special cameras to convert the image into skeleton information [6,7,8,22,24]. Recently, many researchers have developed software-based skeleton extraction systems where OpenPose is one of the most usable systems, which also helps to absorb input noise. Kobayashi et al. extracted geometric features, specifically angle information, which was mainly calculated between adjacent joints, and they achieved 63.6% accuracy using SVM in static signs, which is obtained by their study [10]. Hosoe et al. proposed a static finger spelling recognition system for JSL. They collected a dataset of 5000 samples and applied a 3D articulated hand mode to increase the synthetic 3D image data for training [25]. Finally, they employed the CNN model to classify them to achieve good performance for the JSL recognition system. The main drawback of their work is that they removed dynamic finger spelling, and only the static one is the target. Funasaka et al. used a leap motion controller to collect the dataset for JSL and applied a decision tree and genetic algorithm for the recognition and achieved 74.40% accuracy [26]. Leap Motion Controller is a game controller with software and hardware that can detect hands, fingers, gestures, bars, and motion. To solve the dynamic sign-related problems, Wkatsuki et al. used an infrared TOF Camera to collect the depth camera [27]. They include simple movement dynamic signs, and simple movement means dynamic signs that move in only one direction and do not change the shape of fingers.

Finally, they applied SVM and achieved 90% accuracy based on only simple dynamic fingerspelling, such as moving to left or right and did not include hand shape changing signs such as の (no) or ん (n). Ikuno et al. used a smartphone camera to collect the original data and applied Media-pipe to extract the hand skeleton dataset for JSL. Then, they extracted distance-based explanatory variables as features [28]. Finally, they applied a random forest algorithm and achieved 70% to 80% accuracy in the dynamic sign ‘mo’. Kwolek et al. proposed a JSL recognition system with RGB images. In that work, they followed three steps; firstly, they used a 3D articulated hand model and generative adversarial network (GAN) for generating the synthetic data with graphics techniques to rasterize the photorealistic hand model [29]. After that, they used ResNet34 as a segmentation technique, and finally, they applied an ensemble model to classify the dynamic JSL recognition and achieved 92.10% accuracy. Kobayashi et al. proposed a dynamic finger spelling recognition approach for JSL [30]. They grouped similar dynamic signs such as の (no) and り (ri) and others into the same class. In these rules, there is no difference between の (no) and り (ri). They include dynamic signs with sub-class, which means signs with a similar shape in the same sub-class. They used Open Pose for equipment, MSVM was used, and about 96% accuracy was obtained. Tutui et al. calculated features from coordinates extracted by media-pipe [31]. Finally, they applied SVM as a classifier for the RGB-based JSL and obtained 97.8% accuracy. Ito et al. proposed a CNN to extract features from the JSL dataset, applied SVM for the classification, and achieved 84.20% accuracy [20]. As we know, there are a total of 46 JSL sign alphabets. Among them are considered static signs; five are dynamic or include movement. These five dynamic signs have some moments in which they share the same shape as the example. Figure 1 is an example of dynamic signs in JSL. So, much of Japanese sign language recognition research removes dynamic signs, and their method cannot express these five types of finger spelling that have movement. Most existing systems were developed for static signs by discarding the dynamic alphabet because of the static system. So, much of Japanese sign language recognition research removes dynamic signs, and their method cannot express these five types of finger spelling that have movement. Even if only one character cannot be expressed, many Japanese words become inexpressible and cannot be expressed without using characters represented by dynamic fingers. To overcome the issues, Shiraishi et al. [21] collected the JSL dataset using hand gloves, then applied a machine learning algorithm to classify all 46 Japanese syllabaries, including dynamic signs, and obtained 70% accuracy. Also, they included dynamic signs, but their performance is not good, and they are difficult to deploy in real-life applications. We proposed dynamic Japanese sign language recognition with an effective feature and classification approach to solving the existing challenge. Table 1 demonstrates the literature review summary.

## 3. Dataset Description

We could not locate a dynamic Japanese Sign Language (JSL) dataset online, so we created a new JSL dataset with 20 contributors to address the unavailability of dynamic datasets in the JSL domain. We intend to make this dataset publicly accessible, with the aim of inspiring further research within the JSL community. Additionally, we utilized a Large-scale Argentine Sign Language (LSA) dataset to demonstrate the effectiveness of our proposed model.

### 3.1. Japanese Sign Language(JSL) Dataset

According to researchers, only a few datasets are available for JSL. To address the scarcity of dynamic JSL datasets, we recorded one ourselves using an RGB camera. This dataset comprises consecutive images captured from 20 individuals ranging in age from 11 to 48. Each participant contributed one video for each of the 46 signs, resulting in a total of 920 videos for this dataset. Following standard data collection procedures, we instructed participants to sit in front of the camera, demonstrate a finger-spelling model, and then imitate it. The process took approximately 8 s to capture one character, and each participant spent around 20 min to record all the data. Table 2 provides an overview of the dataset, while Table 3 illustrates the dynamic signs, which vary in the time needed for expression among individuals. As a result, the quantity of data for each dynamic sign differs, whereas the static signs have consistent numbers. For visual reference, Figure 1 showcases examples of both static and dynamic signs. UTF8min

### 3.2. Argentina Sign Language LSA64 Dataset

The Argentina Sign Language Dataset, known as ‘LSA64: A Dataset for Argentinian Sign Language’, comprises 64 distinct signs recorded in the mp4 video format. This dataset encompasses both one-handed and two-handed gestures. Our study is for finger spelling, and this dataset is for sign language. Fingerspelling does not use two hands, and feature generation also assumes one hand. To conduct our analysis, we focused on the 42 one-handed sign language classes, excluding the 22 two-handed signs from the total of 64. In this dataset, there is a total of 3200 data points, as ten individuals performed each of the 64 different signs five times. For validation purposes, we utilized a subset of 2100 data points comprising the 42 one-handed signs; each was performed five times by ten individuals who wore fluorescent gloves while excluding the 22 signs that involved both hands.

## 4. Proposed Methodology

**Figure 2:**
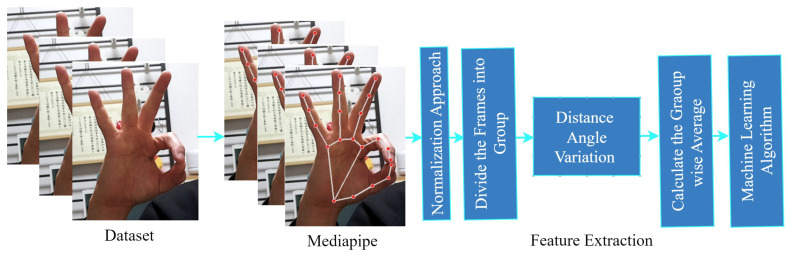
Proposed working flow architecture. The red circle means key-points of the hand that were recognized by mediapipe. Each frame is provided from the video.

The workflow diagram for our proposed study is illustrated in Figure 2. This diagram outlines the key steps we followed in our research. Firstly, we recorded the dataset using an RGB camera. Subsequently, we employed the Mediapipe hand pose detection approach to extract the key points of the hand. Each video in our study consists of approximately 12 frames, and we segmented these frames into four groups. We extracted four types of features from each of these groups using geometrical and mathematical formulas. These features encompass distance, angle, motion, and finger direction. Once we concatenated these features, we applied a machine-learning approach for classification and analysis.

The workflow for our proposed study encompasses the following key steps:

1. **Dataset Acquisition:** We recorded the dataset using an RGB camera to capture Japanese Sign Language (JSL) gestures to address the unavailability of the dynamic JSL dataset.
2. **Hand Pose Detection:** Utilizing the MediaPipe hand pose detection approach, we extracted key points of the hand from the recorded videos.
3. **Frame Segmentation:** Each video in our study comprises approximately 12 frames, which were segmented into four distinct groups for analysis.
4. **Feature Extraction:** From each frame group, we extracted four types of features—distance, angle, motion, and finger direction—employing geometrical and mathematical formulas.
5. **Feature Concatenation:** Concatenating these extracted features, we created a comprehensive feature set that captures both static and dynamic aspects of JSL signs.
  **Optimized Feature Selection and Classification:** Using a feature selection approach based on Random Forest (RF), we identified the most relevant features from the concatenated features, thereby optimizing the efficiency and effectiveness of our model. Additionally, we employed a kernels-based Support Vector Machine (SVM) algorithm for classification.

This methodology aims to enable comprehensive analysis of dynamic JSL gestures, providing a detailed understanding of static and dynamic components through effective feature extraction and machine learning classification.

(1) $$F_{Final}=concate\left[F_{ST},F_{TS},F_{R}\right]$$

### 4.1. Getting Joint Coordinates through Pose Estimation

**Figure 3:**
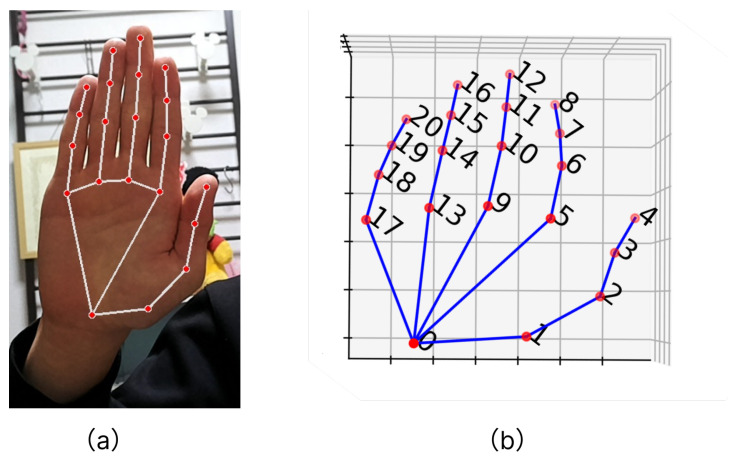
Media-pipe hand pose estimation. (**a**) Rendered Image by Media-pipe system. The red circles mean the key-points that detected by Media-pipe. (**b**) The 3D graph is plotted hand-key-points.

To generate feature values, coordinate data are extracted from images using a hand-shape estimator called MediaPipe [34]. When images containing the hand are processed, they yield x, y, and z coordinate data for 21 landmarks on the hand as real numbers. The landmarks follow a specific order, as illustrated in Figure 3a,b.

### 4.2. Normalization Joint Coordinate Values

Normalization is crucial in skeleton-based datasets for mitigating scale variations. Its primary purpose is to eliminate the impact of scale differences, ensuring equal significance for all joints in modelling tasks. Additionally, normalization enhances the effectiveness and efficiency of the learning process by preventing features with larger scales from dominating. The formula we utilized for normalization is as follows (Equation (2)).

(2) $${x_{i}}^{\prime }=\frac{x_{i}-min\left(x\right)}{max(x)-min(x)}$$

### 4.3. Feature Extraction

**Figure 4:**
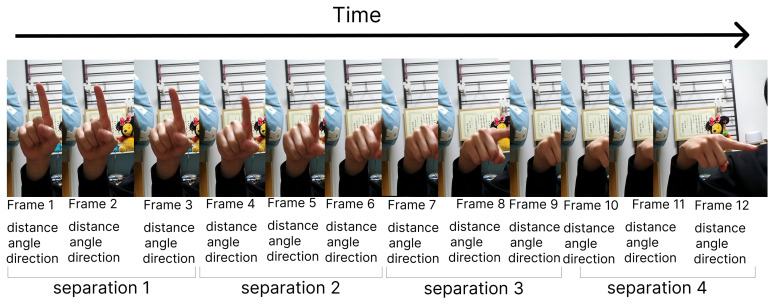
Separate the video frames into groups to calculate feature distance, angle, and finger direction.

To segregate time-oriented behavioural data and the semantic meaning of signs, we partitioned the dataset into quarters. Within each segmentation, we computed features based on rotation type, distance from the palm, angle types derived from fingertips and finger joints, as well as angles and distances relative to the thumb tip. These features aim to capture the unique characteristics of each finger sign. Four distinct feature types are introduced and elaborated upon in this section. To address the variability in the number of image frames used, especially in dynamic finger spelling, we adopted a “Moving Average Calculation” approach during feature extraction. This technique ensures a consistent number of features regardless of the frames used to represent each character. In our implementation, all frames were divided into four segments, and feature values were averaged per segment (as depicted in Figure 4). This “averaging” method was applied to three out of the four features proposed in this study, resulting in feature values representative of the four segments of all frames. For instance, with a total frame count of 12, the segmentation process is completed based on the illustration in Figure 4. In total, 760 distances, 2520 angles, 60 directions, and 189 variations were proposed, leading to a total of 3529 features. However, feature selection was conducted, and it was observed that not all features needed to be utilized to achieve good accuracy in the results. In this section, feature values are extracted from hand coordinate data obtained from RGB image data. The X, Y, and Z coordinates for all 21 hand landmarks are generated by MediaPipe. To mitigate the impact of image size or distance between the hand and the camera, feature values are kept relative. Four distinct types of feature values are proposed in this research, including two feature values derived from Matsuoka’s American sign language recognition with MediaPipe and SVM, specifically “distance” and “angle” [35].

#### 4.3.1. Distance Based Feature

**Figure 5:**
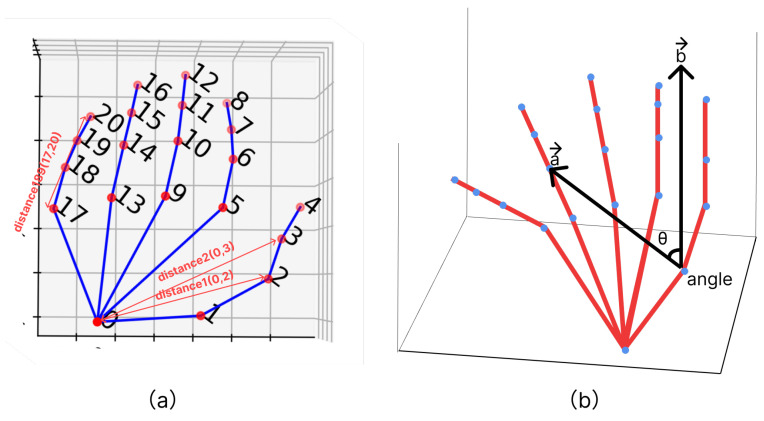
Example of distance and angle calculation procedure. (**a**) Distance feature is calculated the distance between two point except adjacent points. (**b**) Angle feature is calculated between vector $\vec{a}$ that is formed by two key-points and every axis.

The primary feature in our analysis is the relative distance between landmarks, calculated from the 21 hand landmarks. This yields 190 distance feature values for each frame, and with the application of the moving average calculation, we obtain 760 features per dataset. Notably, we exclude the distances between adjacent finger coordinates from our analysis because they remain constant across all hand poses. This decision is based on the understanding that adjacent joint distances do not influence classification, even when hand formations vary significantly. To clarify, Figure 5a illustrates the distance between the 11th and 15th joint points. Although we calculate distances between various joints, we exclude neighboring joint distances as they do not change with hand pose and do not impact classification. Figure 5 provides a visualization of the angle calculation perspective, while Table 4 details each of the distances we compute in our study. The table outlines the starting and ending points for all 190 potential features. It is worth noting that for joints 19 and 20, the distance set is omitted, as other pairs already encompass the expected combinations involving these points. While the distance between joints accounts for variations, challenges related to object size persist. Hand palm size often depends on the individual’s hand size, resulting in variations in distance values. To address this, we employ normalization to mitigate the impact of hand size variations on our analysis.

(3) $$Feature_{Distance}=\sqrt{{\left(x_{i}-x_{j}\right)}^{2}+{\left(y_{i}-y_{j}\right)}^{2}+{\left(z_{i}-z_{j}\right)}^{2}}$$

#### 4.3.2. Angle Based Features

The second feature pertains to the angle between landmarks. Each joint’s angle is calculated using the directed vector of x, y, and z coordinates, reflecting the hand’s tilt based on the direction vector between the 21 landmarks. We generate 210 vectors from these landmarks and calculate angles in each vector’s x, y, and z dimensions. This process results in 630 angle feature values for each segmentation, totaling 2520 features.

Angle features are particularly valuable for distinguishing signs with the same shape but different orientations, such as “ma” and “mi” or “na” and “ni” [20]. Figure 5b illustrates the perspective of angle calculations, while Table 5 provides details on the angles computed in our study. Angle features are especially effective when recognizing signs with identical shapes but varying orientations, like な (na), に (ni), ま (ma), and み (mi). Angle features are more sensitive to such differences and aid in classification [20].

(4) $$cos\left(\theta \right)=\frac{a_{x}\cdot b_{x}+a_{y}\cdot b_{y}+a_{z}\cdot b_{z}}{\sqrt{a_{x}^{2}+a_{y}^{2}+a_{z}^{2}}\cdot \sqrt{b_{x}^{2}+b_{y}^{2}+b_{z}^{2}}}$$

Importantly, angle features are independent of hand size, like distance-based features. This eliminates the impact of the size-related variation. The angle feature calculation formula initially determines the direction vector between two points. Subsequently, it calculates the angle between the vectors using the direction vector and the vector in the X, Y, and Z-axis directions. Let us denote two vectors as $\vec{a}=\left(a_{x},a_{y},a_{z}\right)$ and $\vec{b}=\left(b_{x},b_{y},b_{z}\right)$. The angle feature between these spatial vectors is calculated using Equation (5). To calculate the angle from the X-axis, we set vector $\vec{b}$ as (1, 0, 0) in the X-direction, as expressed in Equation (6). Similarly, the formula for calculating the angle from the Y-axis and Z-axis involves setting vector $\vec{b}$ as (0, 1, 0) and (0, 0, 1) in the Y-axis and Z-axis directions, respectively, as shown in Equations (4)–(7). Table 5 provides an example of angle-based features.

(5) $$cos{\left(\theta \right)}_{x}=\frac{a_{x}}{\sqrt{\left(a_{x}^{2}+a_{y}^{2}+a_{z}^{2}\right)}}$$

(6) $$cos{\left(\theta \right)}_{y}=\frac{a_{y}}{\sqrt{\left(a_{x}^{2}+a_{y}^{2}+a_{z}^{2}\right)}}$$

(7) $$cos{\left(\theta \right)}_{z}=\frac{a_{z}}{\sqrt{\left(a_{x}^{2}+a_{y}^{2}+a_{z}^{2}\right)}}$$

#### 4.3.3. Finger Direction Features

**Figure 6:**
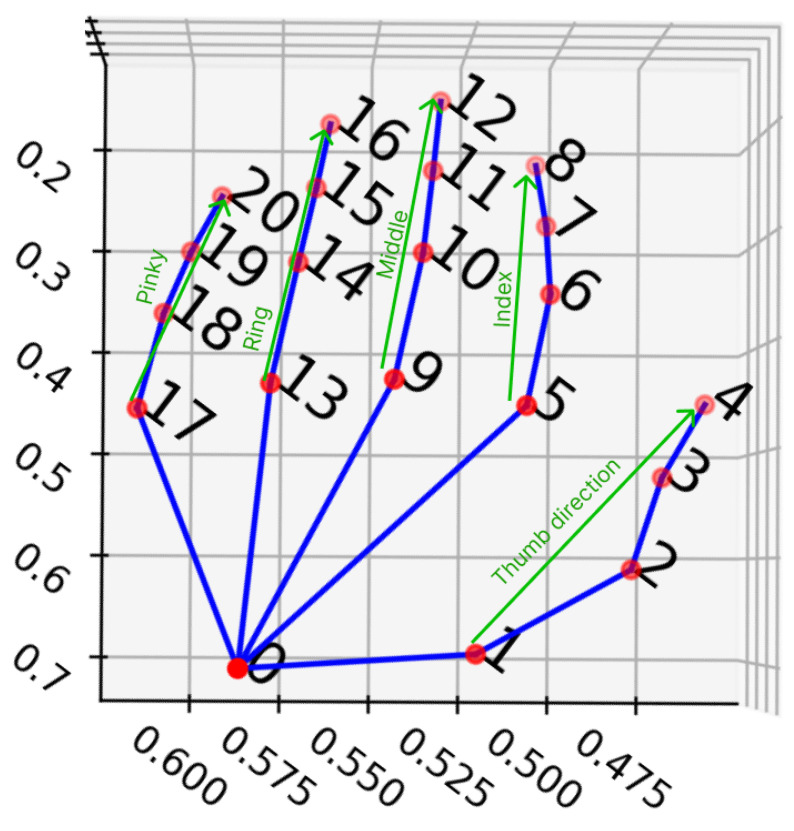
Example of finger direction (green color).

In this feature, the direction of fingers is calculated as a directed x, y, and z vector from the root to the tip of each finger. In fingerspelling, the direction of the finger is important information to improve accuracy, and the moving average calculation method contributes to the recognition of dynamic fingerspelling in the finger direction feature. This feature is also averaged at four separate, and the direction is a Unit vector with direction from fingertip to fingertip, which is visualized in Figure 6 and Equation (8).

(8) $$thumbDirection={landmark}_{4}-{landmark}_{1}$$

#### 4.3.4. Motion Based Features

**Figure 7:**
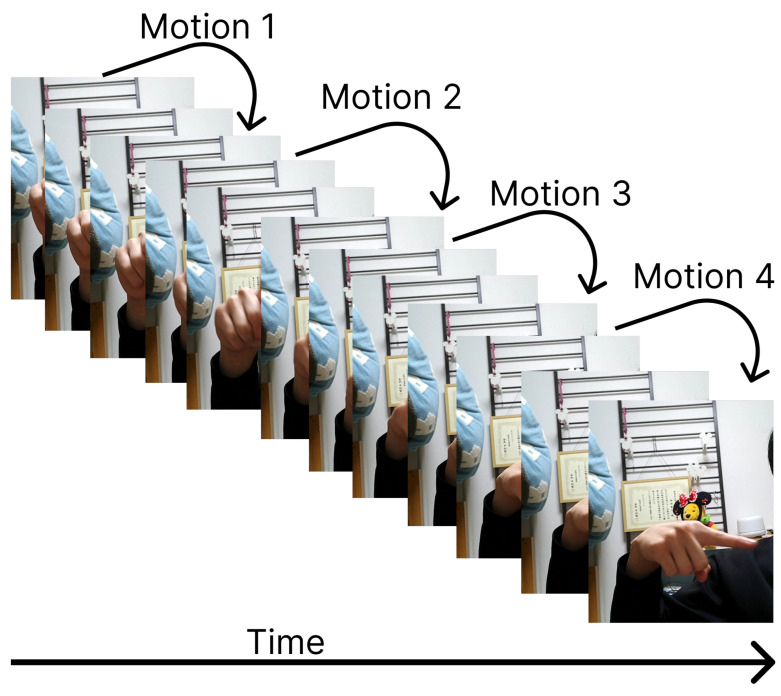
Procedure of Variation Feature.

In our study, we incorporated both static and dynamic signs for JSL despite the fact that many existing research works typically exclude dynamic signs. Motion emerges as a highly effective feature for dynamic gestures. The fourth feature we introduce is the variation in coordinates between frames, essentially representing a directional vector indicating movement that is shown in Figure 7. This feature is integrated to enhance the accuracy of dynamic fingerspelling recognition. The variation amount is computed for each of the 21 landmarks in three dimensions (x, y, and z), resulting in a three-dimensional vector. The calculation procedure for this variation is detailed in Equation (9).

It can be explained in way below Equation (9):

(9) $$Variation=landmark_{ij}-landmark_{(i+frameNum/4)j}$$

### 4.4. Combined All Features for All Frames

There are frames in each video. Each video has a different number of frames, which depends on the time the subject spent on one sign. In the case of static signs, 12 frames were clipped and processed; the number of frames per video data ranged from 4 to 70, with an average of 11.8 frames and one video was divided into four groups. We extracted 190 distance-based features, 630 angle-based features, and 15 direction-based features from a single frame. We averaged the features for three frames and calculated features for four groups for one video. To generate the same number of features for different video lengths, the video data are divided into four equal-time segments and the average of the features generated from the frames within a single segment is used. Suppose the video data have 12 frames; three frames are used per segment. The features generated from those three frames are averaged together to form feature separation. This is conducted for all four separations; 189 variation features do not take the average. In explaining one video, we extracted a total of 190×4+630×4+15×4+189=3529 features.

### 4.5. Feature Selection

We fused the four different features, yielding a large dimension of the feature that can increase the system’s computational complexity. After that, we implemented an RF-based model as a feature selection method to measure the discriminative power of the consistency feature. Feature selection is also an important process because it eliminates features that are not suitable for representing the differences in each sign, resulting in improved accuracy. The RF model mainly generates various classification trees constructed with the bootstrap sample based on the original data. The many trees form a forest, and when a new data trial comes, each tree predicts a result, which is considered a vote for new data. The working procedure of the random forest algorithm is given below:

- It draws a $n_{tree}$ bootstrap samples from the training dataset
- It forms a classification tree from the bootstrap sample. To do this, it randomly selects the predictors’ sample $m_{try}$ and then splits them as the best splitting policies. The tree is grown as much as possible without pruning back. Random forest obtained a predictor for each tree.
- It collects the prediction output of the $n_{trees}$ to predict the new data, then produces the final result using the majority voting system.

In our case, we used the RF for the feature selection. The random forest proved excellent in selecting features with a high dimensional feature nature in many pattern recognition tasks. Chen Lunqin et al. introduced the superiority of RF feature selection in achieving higher accuracy using fewer features and comparing the final accuracy to other algorithms by evaluating the importance of features using RF [36]. In our case, we extracted 3529 features which also have a high dimensional feature nature. The main purpose of selecting features for two issues is (i) to improve the model performance by avoiding overfitting problems and (ii) to explore the internal underlying process of the generated data. A random forest approach was performed in the feature selection task, unlike other classification feature selection methods, such as SFFS or Boruta. Permutation importance index (PIM) and Gini importance index (GII) are the two most commonly used for potential variable measurement. GII first calculates the Gini index and then measures the node impurity. The sum of the Gini index reduction in all nodes generated the feature importance value for the single tree. The Gini index average or summation of all trees in the forest conveys the overall variable importance. However, PIM is the most popular technique used to select the potential feature using RF. To construct individual trees, RF does not use all the datasets at a time and randomly selects some of the data and leaves set out of bags (OOB). To measure the specific feature importance in a tree. Here, is a graph plotting the number of features we used out of the 3529 features we devised on the x-axis and the accuracy of the model using those features on the y-axis. To explain the numbers in the graph, when the rf feature importance is added in order from the top, it reaches 90% using only 13 features and then 95% using 25 of these features. The greatest accuracy was recorded in the model using 388 features, with an accuracy of 0.971.

The mathematical explanation of the RF feature selection algorithm is given in [36] and is as follows: A decision tree with *M* leaves divides the feature space into *M* regions Rm, 1≤m≤M. For each tree, the prediction function f(x) is defined as (10).

(10) $$\begin{array}{c} f\left(x\right)=\sum_{m=1}^{M}C_{m}\prod \left(x,R_{m}\right) \end{array}$$

### 4.6. Classification Module

In the study, a well-known supervised machine learning with various tuning parameters, namely the Support vector machine (SVM) that we used as a classifier [37]. The main idea of SVM is to produce a hyperplane that aims to divide the data points into different classes based on the various feature spaces with a large margin and maximize this margin as much as possible. In the case of a two-dimensional feature, it generates a line, and for the high-dimensional data, it generates some sub-space. To search for the optimal hyperparameters of the SVM model, we used to leverage Optuna’s optimization capabilities [38]. We defined the objective function, which is used to optimize Optuna; the objective function trained the use of the SVM model with the specific range of hyperparameters and ultimately generated a cross-validated mean accuracy. The optimization process was conducted by Optuna by exploring different combinations of hyperparameters using the search space. The range of the specific parameters is given in Table 6. There are four types of kernels that were experimented on in this research (poly, linear, rbf, and sigmoid).

**Figure 8:**
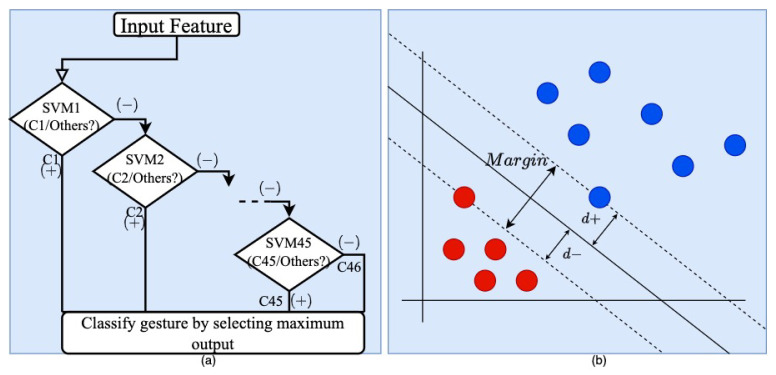
Example of (**a**) OVR mechanism of SVM (**b**) Binary class classification of SVM.

SVM is used for machine learning classification and is also used for hand pose recognition in [39] by Phat Nguyen Huu and Tan Phung Ngoc. The training will compute the support vector that will divide the 46 labels this time. To classify the 46 labels in this study, we used a multiclass SVM as in [40]. We use the usual binary classification SVM and introduce (OVR) for classifying one versus the other and assign the class classified with the highest output function. By repeating this process, the SVM classified 46 classes. As shown in Figure 8a,b below, the OVR SVMs operated in parallel, separating one class from the others and measuring the largest one.

(11) $$\begin{array}{ccc} f_{i}\left(x\right) & = & w_{i}^{t}x+b_{i} \end{array}$$

(12) $$\begin{array}{ccc} X & \mapsto & arg\max_{i}f_{i}\left(x\right) \end{array}$$

The following figure shows an example of binary classification and the multi-class classification OVR SVM approach.

## 5. Experimental Performance

To assess the proposed model, we utilized our own dataset, evaluating its performance across various scenarios by extracting different types of features using mathematical and geometrical formulas.

### 5.1. Environmental Setting

We extracted a total of 1195 features from different parts of the hands and applied min-max normalization to these features. Subsequently, we employed a support vector machine with a radial basis function for model training, utilizing 5-fold cross-validation on the training set to compute identification accuracy. This process was repeated five times, and we calculated the average identification accuracy for robust evaluation. The implementation of our model was carried out in a Python environment, leveraging various statistical and geometrical Python packages. This experiment was conducted on a GPU PC that is supplied by FRONTIER (BTO PC maker), Yokohama, Kanagawa prefecture, Japan with an Intel^®^ Core^™^ i9 13900K CPU, 64 GB RAM and an NVIDIA^®^ Geforce RTX^™^ 4090.

### 5.2. Ablation Study

The new idea in the study is to divide the sequential frame into four groups to increase feature effectiveness in terms of the finger configuration features. We also experimented with the proposed method by dividing sequential frames into 1 to 4 for both the JSL and LSA64 SL datasets, revealing differences in accuracy. Since the number of features varies with the number of divisions, the following table summarizes the differences in the number and result of features generated for each of the different numbers of divisions. In this table, the selected features represent the number of features chosen by the RF algorithm, and the accuracy is the result of applying SVM and other four deep learning algorithms, including LSTM, Bi-LSTM, Two-Stream GRU and Two Stream-BiGRU using the selected features.

However, keep in mind that deep learning experiments are slightly different from machine learning experiments. Specifically, the deep learning model used in the Ablation Study is a sequence analysis model, so the sequence length of the input changes depending on the number of divisions because we considered the division to be in sequence. For example, the input shape when divided into three will be (3, Num_of_Features). In addition, the features of “Distance”, “Angle”, and “Direction” are generated for each division, but the “Variation” we propose is the amount of change between divisions, so the sequence length of “Variation” is one less than the sequence length of other features. Therefore, the Deep Learning model has become a two-stream model.

For the JSL dataset, three divisions were better than four, and for the LSA64 dataset, four divisions were better. The simpler JSL dataset is likely superior because only five types of data are dynamic, while the LSA64 dataset is better because all data are dynamic.

Table 7 presents the results of the ablation study for the proposed model, which systematically investigates the impact of varying divisions on the recognition performance across two distinct datasets: Japanese Sign Language (JSL) and LSA64. In the case of the JSL dataset, with each increasing division from 1 to 4, the recognition accuracy exhibits a steady improvement, reaching its peak at Division 3 with an accuracy of 97.20%. This suggests that a more detailed segmentation of sequential frames enhances the model’s ability to capture the intricacies of both static and dynamic signs. Conversely, for the LSA64 dataset, Division 4 yields the highest accuracy of 98.40%, with Division 3 closely following at 98.00%. This trend implies that the dynamic nature of all signs in the LSA64 dataset benefits from a more detailed division, resulting in superior recognition accuracy. We also perform well with the proposed feature using a deep learning algorithm, but SVM performance accuracy is near or higher than that of the deep learning method. SVM uses the CPU, while deep learning uses the GPU. In this experimental environment, the GPU is about 45 times faster than the CPU for the two devices, but the computation time is almost the same for SVM and deep learning, and the lack of restrictions imposed by the need for a GPU is an important factor in the potential of this research for applied research and practical applications. Therefore, we compared both methods; the SVM model was chosen as our main module. The findings emphasize the significance of tailoring division strategies to the specific characteristics of the analyzed sign language, ultimately optimizing the model’s performance for diverse datasets.

### 5.3. Performance Accuracy with Proposed JSL Dataset

We extracted four types of features: Distance, Angle, Direction, and Variation. To assess their individual impact on accuracy, we conducted experiments using each feature type separately. The results are presented in Table 8. Notably, the Angle feature achieved the highest accuracy at 95%, followed by DISTANCE at 93%. Variation yielded a much lower accuracy because most Japanese signs are static, and Variation captures hand motions, making it less relevant for the majority of signs. The table also includes results when all features were used, resulting in an accuracy of 94%, slightly lower than using just the Angle feature. It is possible that not all features are equally suitable for classification. Therefore, the next section (Feature Selection) focuses on identifying optimal combinations of these features to enhance accuracy.

To demonstrate the impact of the variation feature, we experimented with the proposed model with two datasets, including the variation feature and without including the variation feature. Table 9 compares the performance accuracy of two datasets, JSL and LSA64, in the presence and absence of variation features. For the JSL dataset, the accuracy rises from 95.61% without variation to 97.20% with variation, emphasizing the positive impact of incorporating variation features. Similarly, the LSA64 dataset experiences an accuracy increase from 97.77% to 98.40% when variation features are introduced. The number of selected features also varies between the scenarios, with a reduction observed in both datasets when variation features are included. These results suggest that the inclusion of variation features enhances the accuracy of the datasets, indicating the importance of considering variations in feature selection for improved performance in the respective contexts of the JSL and LSA64 datasets. In addition, Table 10 demonstrated the label wise classification accuracy for the JSL alphabet dataset which demonstrated that 100% accuracy for the 41 labels and more than 85% accuracy for the one labels and 70–80% accuracy for rest 5 labels.

#### 5.3.1. Optimal Feature Based Performance Analysis of the Proposed JSL Dataset

The experiment was repeated by increasing the number of features used one by one, starting with the highest-ranked of all the features. Every time, the combination of features was fed into an SVM-based classifier, and each trail’s identification accuracy was computed. After that, the average identification accuracy was computed for each combination. We observed that our proposed system produced higher performance scores for combining 493 features for arbitrary dynamic hand gesture patterns than the same dynamic hand gesture pattern. We extracted four features in the study: averaging the distance, angle, finger direction and non-averaged variation. In this research, a variation feature was added to improve the accuracy of dynamic sign recognition. As a variation feature, we are applying a new feature, JSL classification; we need to visualize the impact of the variation feature for performance accuracy. Moving average calculation was also applied to distance, angle, and direction to recognize dynamic fingerspelling.

#### 5.3.2. State of the Art Similar Work Comparison for the JSL Dataset

The proposed model’s similar work state-of-the-art comparison performance is demonstrated in Table 11. The comparison is performed with similar work, but a difference was extracted, and a total of 1195 features from different parts of the hands were applied with min-max normalization applied to these features. Subsequently, we trained a support vector machine with a radial basis function using 5-fold cross-validation on the training set to compute identification accuracy. We repeated This process five times and calculated the average identification accuracy. The comparison performance table visualized that the performance accuracy of the proposed model is higher than the existing performance. Among the existing state-of-the-art work, Ikuno et al. used a smartphone camera to collect the original data. They applied Media-pipe to extract the hand skeleton dataset for JSL. Then, they extracted distance-based explanatory variables as features, and they achieved 70–80% accuracy with random forest [28]. Kwolek et al. proposed three steps to recognize the JSL alphabet. First, they used a 3D articulated hand model and generative adversarial network (GAN) for generating the synthetic data, then used ResNet34 as a segmentation technique, and finally, they applied an ensemble model to classify the dynamic JSL recognition and achieved 92.10% accuracy [29]. Kobayashi et al. grouped similar dynamic signs such as の (no) and り (ri) and others into the same class; they used subclass and sub-class means signs that have similar shapes into the same sub-class for the dynamic sign [30]. The equipment utilized in their study involved the use of Open Pose, and for classification, they employed MSVM, which yielded an accuracy of approximately 96.00%. In contrast, our proposed model achieved a higher accuracy of 97.20% when compared to this existing system. This improvement in accuracy underscores the effectiveness of our innovative approach, demonstrating its superiority over established methods in the domain of sign language recognition.

### 5.4. Performance Accuracy with Public LSA64 Dataset

There is probably no Japanese sign language dataset, yet that includes dynamic finger spelling. However, it is important to use public data to prove the generality of our features. Therefore, we try datasets that publish hand gestures, but not limited to Japanese Sign Language or fingerspelling, to prove generalization. Since this dataset is not Japanese Sign Language, it does not validate models trained for static and dynamic signs since all the data are signs with motion. However, since there are only five types of Japanese Sign Language, we think that this dataset can be used to show that our features are effective for dynamic signs. Table 12 demonstrated the label-wise performance for the LSA64 dataset. On average, for all datasets, our model achieved 98.20% accuracy. By observing the performance accuracy of the proposed model with the existing dataset, we can say that our proposed method proved its superiority and effectiveness for dynamic hand gesture recognition.

### 5.5. Discussion

The results in the “Results” section demonstrate that adding the Variation feature led to improved accuracy in dynamic Japanese sign language recognition. This feature proved particularly effective, reducing prediction errors significantly, especially for signs like お (o) and を (wo), where errors decreased from twelve to just one. Overall, dynamic accuracy saw a substantial increase, improving from 91.6% to 98.4%. Interestingly, the inclusion of the Variation feature had only a minor impact on overall accuracy, likely because there are only five dynamic signs in the dataset. Furthermore, the Variation feature contributed to fewer prediction errors for signs like て (te) and け (ke), which have similar hand shapes. This improvement can be attributed to the feature’s ability to capture subtle changes in hand movements, such as the flexion of the thumb, which can affect the sign’s interpretation.

## 6. Conclusions

Our study introduces an innovative approach to dynamic Japanese Sign Language (JSL) recognition, leveraging effective feature extraction and classification techniques. Through meticulous data collection using a comprehensive video dataset and precise coordinate extraction with MediaPipe, we proposed four distinct features from each group of the sequential frames that are crucial for dynamic JSL recognition. These optimized features were integrated into a classification algorithm, challenging the traditional exclusion of dynamic signs in JSL recognition studies. Historically, dynamic signs were often omitted due to their lower accuracy; however, our research achieved remarkable accuracy in both static and dynamic cases, showcasing the superiority of our model. Notably, our model demonstrated exceptional accuracy using only two frames for five dynamic signs, highlighting its potential for recognizing all 46 Japanese sign language dynamic signs with high precision. Our proposed model empowers individuals to convey a wide array of Japanese words through JSL recognition, mirroring the proficiency of JSL experts. Our future plans involve the development of a real-time JSL recognition system encompassing an alphabet and vocabulary, enhancing practical utility and accessibility. This research marks a significant milestone in advancing effective and inclusive communication through Japanese Sign Language (JSL). Looking ahead, we plan to extend our efforts to JSL word recognition to further enhance the capabilities of the proposed system. Our commitment to ongoing development reflects our dedication to providing comprehensive solutions that contribute to the accessibility and richness of communication for the deaf and hard-of-hearing community using JSL.

## Figures and Tables

**Table 1:** Summary of the existing work.

Reference	Equipment	Classifier	Accuracy	Target	Years
[32]	OpenPose	SVM	63.6	static	2019
[20]	RGB Camera	CNN and SVM	84.7	static	2019
[33]	RGB Camera	CNN and SVM	86.2	static	2020
[21]	Data Glove	CNN and SVM	70	static + dynamic (all)	2020
[25]	RGB Camera	CNN	93	static	2017
[26]	LeapMotion	decision tree	74.7	static	2015
[32]	Infrared TOF	SVM	90	static + dynamic	2013
[28]	RGB Camera	random forest	79.2	static	2021
[29]	RGB Camera	deep CNN	92.1	static	2021
[30]	OpenPose	MSVM	65.6 96	static + dynamic (subclass)	2019
[31]	RGB Camera	SVM	97.8	static	2022

**Table 2:** Proposed dataset description.

Dataset Name	Classes	Recording Time 1 p/s	Average Frames	People	Age Range	Total Frames
JSL	46 あ [a] ∼ん [n]	2.00 s	12	20	11∼48	11,040

**Table 3:** Average time in each dynamic sign.

Name	42 (の [no])	43 (も [mo])	44 (り [ri])	45 (を [wo])	46 (ん [n])
Frame average	14.75	8.05	10.2	9.55	9.37
Average seconds	3.20	1.74	2.21	2.1	2.029

**Table 4:** Considered set of starting and ending joint points for calculating distance-based features.

Starting Joint Number	Distance to the Joint Numbers	Number of Distance Based Features
0	{2,3,5,6,7,9,10,11,13,14,15,17,18,19,20}	15
1	{3,4,5,6,7,8,9,10,11,12, 13,14,15, 16,17,18,19,20}	18
2	{4,5,6,7,8,9,10,11,12, 13,14,15, 16,17,18,19,20}	17
3	{5,6,7,8,9,10,11,12, 13,14,15, 16,17,18,19,20}	16
4	{5,6,7,8,9,10,11,12, 13,14,15, 16,17,18,19,20}	16
5	{7,8,9,10,11,12, 13,14,15, 16,17,18,19,20}	14
6	{8,9,10,11,12, 13,14,15, 16,17,18,19,20}	13
7	{9,10,11,12, 13,14,15, 16,17,18,19,20}	12
8	{9,10,11,12, 13,14,15, 16,17,18,19,20}	12
9	{11,12, 13,14,15, 16,17,18,19,20}	10
10	{12, 13,14,15, 16,17,18,19,20}	9
11	{13,14,15, 16,17,18,19,20}	8
12	{13,14,15, 16,17,18,19,20}	8
13	{15, 16,17,18,19,20}	6
14	{16,17,18,19,20}	5
15	{17,18,19,20}	4
16	{17,18,19,20}	4
17	{19,20}	2
18	{20}	1
19	{}	0
20	{}	0

**Table 5:** Set of vectors obtained when each joint point is considered as a starting point for measuring angle-based features.

Joint Index	Starting Joint (P) Variable Joint (V)	Other Joints (V)	Features
0	$\vec{P_{0}V}$	$P_{1},P_{2},P_{3},P_{4},P_{5},P_{6},P_{7}$ $P_{8},P_{9},P_{10},P_{11}P_{12},P_{13},P_{14}$ $P_{15},P_{16},P_{17},P_{18},P_{19},P_{20}$	20 × 3
1	$\vec{P_{1}V}$	$P_{2},P_{3},P_{4},P_{5},P_{6},P_{7}$ $P_{8},P_{9},P_{10},P_{11}P_{12},P_{13},P_{14}$ $P_{15},P_{16},P_{17},P_{18},P_{19},P_{20}$	19 × 3
2	$\vec{P_{2}V}$	$P_{3},P_{4},P_{5},P_{6},P_{7}$ $P_{8},P_{9},P_{10},P_{11}P_{12},P_{13},P_{14}$ $P_{15},P_{16},P_{17},P_{18},P_{19},P_{20}$	18 × 3
3	$\vec{P_{3}V}$	$P_{4},P_{5},P_{6},P_{7}$ $P_{8},P_{9},P_{10},P_{11}P_{12},P_{13},P_{14}$ $P_{15},P_{16},P_{17},P_{18},P_{19},P_{20}$	17 × 3
4	$\vec{P_{4}V}$	$P_{5},P_{6},P_{7}$ $P_{8},P_{9},P_{10},P_{11}P_{12},P_{13},P_{14}$ $P_{15},P_{16},P_{17},P_{18},P_{19},P_{20}$	16 × 3
5	$\vec{P_{5}V}$	$P_{5},P_{6},P_{7}$ $P_{8},P_{9},P_{10},P_{11}P_{12},P_{13},P_{14}$ $P_{15},P_{16},P_{17},P_{18},P_{19},P_{20}$	15 × 3
6	$\vec{P_{6}V}$	$P_{7},P_{8},P_{9},P_{10},P_{11}P_{12},P_{13}$ $P_{14}$ $P_{15}$, $P_{16}$, $P_{17}$, $P_{18}$, $P_{19}$, $P_{20}$	14×3
…	…	…	…
19	$\vec{P_{19}V}$	$P_{20}$	1×3
20	$\vec{P_{20}V}$		0

**Table 6:** The range of hyper-parameters.

SVM Parameters	Range
C	$1\times 10^{0}\approx \frac{1\times 10^{2}}{2}$
Gamma	$1\times 10^{-3}\approx 3.0$
Degree	1≈10
Trial	300

**Table 7:** Ablation study of the proposed model. CPU: Intel^®^ Core^™^ i9 13900K (about 1.8 TFLOPS). GPU: NVIDIA^®^ GeForce RTX^™^ 4090 (about 82.58 TFLOPS).

Dataset Name	Division	Total Feature	Input Shape	Classification Algorithm	Accuracy	Computation Time in [ms]	Device
JSL	1	898	128	SVM	96.00	0.23	CPU
JSL	2	1796	208	SVM	96.80	0.27	CPU
JSL	2	1796	(2, 835), (2, 63)	Two-Stream LSTM	97.30	0.16	GPU
JSL	2	1796	(2, 835), (2, 63)	Two-Stream Bi-LSTM	97.20	0.19	GPU
JSL	2	1796	(2, 835), (2, 63)	Two-Stream GRU	96.99	0.15	GPU
JSL	2	1796	(2, 835), (2, 63)	Two-Stream Bi-GRU	97.14	0.17	GPU
JSL	3	2631	493	SVM	97.20	0.34	CPU
JSL	3	2631	(3, 835), (2, 63)	Two-Stream LSTM	97.34	0.16	GPU
JSL	3	2631	(3, 835), (2, 63)	Two-Stream Bi-LSTM	97.28	0.19	GPU
JSL	3	2631	(3, 835), (2, 63)	Two-Stream GRU	97.07	0.15	GPU
JSL	3	2631	(3, 835), (2, 63)	Two-Stream Bi-GRU	97.13	0.17	GPU
JSL	4	3529	550	SVM	97.00	0.37	CPU
JSL	4	3529	(4, 835), (3, 63)	Two-Stream LSTM	97.09	0.17	GPU
JSL	4	3529	(4, 835), (3, 63)	Two-Stream Bi-LSTM	97.19	0.21	GPU
JSL	4	3529	(4, 835), (3, 63)	Two-Stream GRU	96.79	0.15	GPU
JSL	4	3529	(4, 835), (3, 63)	Two-Stream Bi-GRU	96.99	0.18	GPU
LSA64	3	2631	341	SVM	98.00	0.28	CPU
LSA64	3	2631	(3, 835), (2, 63)	Two-Stream LSTM	97.75	0.16	GPU
LSA64	3	2631	(3, 835), (2, 63)	Two-Stream Bi-LSTM	97.51	0.19	GPU
LSA64	3	2631	(3, 835), (2, 63)	Two-Stream GRU	97.62	0.15	GPU
LSA64	3	2631	(3, 835), (2, 63)	Two-Stream Bi-GRU	97.60	0.17	GPU
LSA64	4	3529	326	SVM	98.40	0.27	CPU
LSA64	4	3529	(4, 835), (3, 63)	Two-Stream LSTM	97.38	0.19	GPU
LSA64	4	3529	(4, 835), (3, 63)	Two-Stream Bi-LSTM	97.53	0.23	GPU
LSA64	4	3529	(4, 835), (3, 63)	Two-Stream GRU	97.55	0.16	GPU
LSA64	4	3529	(4, 835), (3, 63)	Two-Stream Bi-GRU	97.51	0.18	GPU

**Table 8:** Accuracy with individual features.

Feature	Number of Features	Accuracy
Distance	760	93.00
Angle	2520	95.00
Direction	60	89.00
All features	3529	96.26

**Table 9:** Performance accuracy with variation features.

Dataset	Without Variation		With Variation		SVM Tunned Parameter
	**Number of Selected Feature**	**Accuracy**	**Number of Selected Feature**	**Accuracy**	
JSL	421	95.61	367	97.20	Kernel = rbf, C = 24.61, Gamma = 0.0015
LSA64	209	97.77	326	98.40	Kernel = rbf, C = 24.61, Gamma = 0.0015

**Table 10:** Labelwise Performance result with JSL.

No.	Labels	Accuracy	No.	Labels	Accuracy
1	あ (a)	1.00	24	ね (ne)	1.00
2	い (i)	1.00	25	の (no)	1.00
3	う (u)	1.00	26	は (ha)	1.00
4	え (e)	1.00	27	ひ (hi)	1.00
5	お (o)	75.00	28	ふ (he)	1.00
6	か (ka)	1.00	29	へ (he)	86.00
7	き (ki)	1.00	30	ほ (ho)	1.00
8	く (ku)	1.00	31	ま (ma)	1.00
9	け (ke)	1.00	32	み (mi)	1.00
10	こ (ko)	71.00	33	む (mu)	1.00
11	さ (sa)	1.00	34	め (me)	1.00
12	し (si)	1.00	35	も (mo)	1.00
13	す (su)	1.00	36	や (ya)	75.00
14	せ (se)	1.00	37	ゆ (yu)	1.00
15	そ (so)	1.00	38	よ (yo)	1.00
16	た (ta)	1.00	39	ら (ra)	1.00
17	ち (ti)	1.00	40	り (ri)	1.00
18	つ (tu)	1.00	41	る (ru)	1.00
19	て (te)	1.00	42	れ (re)	1.00
20	と (to)	1.00	43	ろ (ro)	1.00
21	な (na)	1.00	44	わ (wa)	1.00
22	に (ni)	1.00	45	を (wo)	75.00
23	ぬ (nu)	1.00	46	ん (n)	1.00

**Table 11:** State of the art-similar work comparison of the proposed model with JSL Dataset.

Reference	Recording Device	Features	Algorithm Name	Result Score [%]	Type of Sign
Ikuno et al. [28]	RGB Camera		random forest	79.20	static
Kwolek et al. [29]	RGB Camera		deep CNN	92.10	static
Kobayashi et al. [30]	Open Pose		MSVM	65.00 96.00	static + dynamic
Proposed method	Camera	Distance, Angle, Finger Direction, Motion	SVM	97.20	Static + dynamic

**Table 12:** Labelwise Performance result with Argentina LSA64 dataset.

No.	Labels	Accuracy	No.	Labels	Accuracy
1	Opaque	1.00	22	Water	1.00
2	Red	1.00	23	Food	91.00
3	Green	1.00	24	Argentina	1.00
4	Yellow	1.00	25	Uruguay	1.00
5	Bright	1.00	26	Country	1.00
6	Light-blue	90.00	27	Last name	1.00
7	Colors	1.00	28	Where	83.00
8	Pink	1.00	29	Birthday	1.00
9	Women	1.00	30	Hungry	92.00
10	Enemy	1.00	31	Ship	1.00
11	Son	1.00	32	None	1.00
12	Man	83.00	33	Name	1.00
13	Away	1.00	34	Patience	1.00
14	Drawer	1.00	35	Perfume	1.00
15	Born	89.00	36	Deaf	1.00
16	Learn	1.00	37	Candy	1.00
17	Call	1.00	38	Chewing-gum	1.00
18	Skimmer	1.00	39	Shut down	1.00
19	Bitter	1.00	40	Buy	1.00
20	Sweet milk	1.00	41	Realize	79.00
21	Milk	1.00	42	Find	1.00

## Data Availability

LSA64 Dataset is available in the below link: http://facundoq.github.io/datasets/lsa64/ (accessed on 22 December 2023).

## Abbreviations

JSL	Japanese Sign Language
PCA	principal component analysis
HMM	Hidden Markov model
ANN	Artificial neural networks
FCA	Fuzzy classification algorithms
KNN	K-nearest-neighbor
CNN	Convolutional Neural Network
ASL	American Sign Language
